# Circulating autophagy regulator Rubicon is linked to increased myocardial infarction risk

**DOI:** 10.1016/j.jmccpl.2024.100279

**Published:** 2024-12-21

**Authors:** Marie-Hélène Grazide, Jean-Bernard Ruidavets, Wim Martinet, Meyer Elbaz, Cécile Vindis

**Affiliations:** a Center for Clinical Investigation (CIC1436)/CARDIOMET, Rangueil University Hospital, Toulouse, France; b University of Toulouse III, Toulouse, France; c Department of Epidemiology, INSERM UMR 1027, Toulouse, France; d Laboratory of Physiopharmacology, University of Antwerp, Antwerp, Belgium; e Department of Cardiology, Rangueil University Hospital, Toulouse, France

**Keywords:** Rubicon, Autophagy, Biomarker, Myocardial infarction, Risk prediction

## Abstract

**Background:**

The identification of new biomarkers that improve existing cardiovascular risk prediction models for acute coronary syndrome is essential for accurately identifying high-risk patients and refining treatment strategies. Autophagy, a vital cellular degradation mechanism, is important for maintaining cardiac health. Dysregulation of autophagy has been described in cardiovascular conditions such as myocardial ischemia-reperfusion injury, a key factor in myocardial infarction (MI). Recently, Rubicon (Run domain Beclin-1-interacting and cysteine-rich domain-containing protein), a key negative regulator of autophagy, has been identified in the modulation of cardiac stress response.

**Objectives:**

This study aimed to explore the relationship between circulating Rubicon levels and MI, and to evaluate the incremental predictive value of Rubicon when integrated into a clinical risk prediction model for MI.

**Results:**

We analyzed plasma Rubicon concentrations in 177 participants, comprising type I MI patients and high-risk control subjects. Our results revealed significantly elevated plasma Rubicon levels in MI patients compared to the control group (126.5 pg/mL vs. 53 pg/mL, *p* < 0.001). Furthermore, Rubicon levels showed a positive correlation with cardiovascular risk factors such as total cholesterol and LDL cholesterol. Multivariate analysis confirmed that Rubicon levels were independently associated with an increased risk of MI. The inclusion of Rubicon in traditional cardiovascular risk models notably enhanced predictive accuracy for MI, with the area under the curve (AUC) rising from 0.868 to 0.905 (*p* < 0.001).

**Conclusions:**

These findings suggest that Rubicon is a valuable biomarker associated with MI risk, providing additional predictive value beyond standard cardiovascular risk factors. This highlights the importance of Rubicon's critical role in the pathophysiology of CVD.

## Highlights

• Elevated Rubicon levels are significantly associated with myocardial infarction (MI).
• Rubicon correlates positively with cholesterol levels, including total and LDL cholesterol.
• Rubicon independently predicts MI risk, adding value to cardiovascular risk models.
• Adding Rubicon to risk models improves MI prediction accuracy (AUC increased from 0.868 to 0.905).

## Graphical abstract

**Figure:**
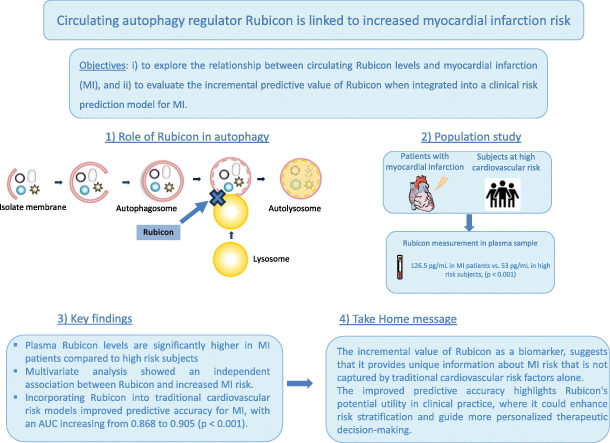

## 1 Introduction

Cardiovascular disease (CVD) remains a leading cause of global morbidity and mortality [1]. Despite significant advancements in the acute management and secondary prevention of acute coronary syndrome, the overall burden of CVD is expected to rise due to the aging population and increasing incidence of conditions such as obesity and diabetes. In light of this, there is a continuing need for new biomarkers that can enhance risk assessment and guide therapeutic decisions for patients with CVD.

Autophagy, a fundamental cellular mechanism for degrading and recycling damaged organelles and proteins through lysosome-mediated pathways, plays a significant role in maintaining cellular health [2]. This process involves the formation of double-membrane structures, known as autophagosomes, that encapsulate cytoplasmic material and deliver it to lysosomes for degradation. Autophagy is essential for maintaining cellular homeostasis, and dysregulation of this process has been implicated in various diseases, including cardiovascular conditions such as myocardial ischemia-reperfusion (I/R) injury [[3], [4], [5]]. This condition is a known contributor to type 1 myocardial infarction (MI), which results from atherosclerotic plaque rupture, a hallmark of both ST-elevation MI (STEMI) and non-ST-elevation MI (NSTEMI), typically grouped under ACS [6]. Once activated, autophagy progresses through four sequential steps, each of which requires specific regulatory proteins and complexes. These steps have been extensively described in several reviews and will not be detailed here [7].

In response to ischemic injury, autophagy is activated in cardiomyocytes, where it serves as an adaptive mechanism to alleviate metabolic stress and promote cell survival [[8], [9], [10]]. However, excessive autophagy can lead to a newly recognized form of cell death termed autosis, particularly during the late reperfusion phase following I/R injury [11]. Notably, this process is characterized by the upregulation of Rubicon (RUN domain Beclin-1 interacting and cysteine-rich-containing protein), a key inhibitor of autophagosome maturation and autophagosome-lysosome fusion [12,13].

Beyond its role in autophagy regulation, Rubicon is involved in other cellular processes such as LC3-associated phagocytosis (LAP), endosomal trafficking, and the regulation of inflammation [[14], [15], [16]]. Given its close association with myocardial I/R injury, Rubicon represents a promising candidate as both a biomarker for autophagy activity and a therapeutic target for modulating autophagy in clinical settings [11,17].

Despite the critical role of autophagy in cardiac pathology, the use of circulating autophagy-related proteins as accessible biomarkers in cardiovascular pathology remains underexplored. Using available immunoassay technologies, a panel of autophagy-related proteins has been successfully measured in human body fluids. Previous studies have demonstrated that circulating levels of autophagy-related proteins, such as ATG5 and Parkin, may serve as diagnostic markers in conditions such as cognitive decline and hypoxic-ischemic encephalopathy [18,19]. Additionally, circulating levels of Beclin1 have been linked to disease severity in chronic obstructive pulmonary disease (COPD) [20] and are elevated in long-lived individuals, including centenarians, compared to younger populations or those with MI [21].

Recently, our research demonstrated an inverse relationship between plasma Rubicon levels and the risk of acute coronary syndrome (ACS) in patients experiencing their first event [22]. In this study, we sought to further validate these findings by measuring plasma Rubicon levels in a different cohort, including patients with acute myocardial infarction (MI) and asymptomatic individuals at very high risk, as assessed by the Systematic Coronary Risk Evaluation (SCORE) model [23]. We also explored the association between Rubicon levels and myocardial infarction, along with its predictive power for MI.

## 2 Materials and methods

### 2.1 Study population

The study population consisted of 177 subjects admitted to the Department of Interventional Cardiology and Prevention of Cardiovascular Disease at Toulouse University Hospital Center in Toulouse, France. This case–control study included 79 patients (men and women aged >18 years) diagnosed with acute type I MI, admitted within 48 h of symptom onset, and consecutively recruited between November and December 2015 (DC-2008-4623) [24]. Blood samples were collected within 2 to 3 days after the acute ischemic event. The control group consisted of 98 subjects (men and women) from a previously described case–control study (NCT02405468) [25]. These control subjects were at high cardiovascular risk for primary prevention, defined by the presence of at least two of the following risk factors: treated dyslipidemia, treated hypertension, treated diabetes mellitus, or current smoking. The control group consisted of individuals with a high risk of cardiovascular events who were undergoing primary prevention measures. High risk was determined by the presence of at least two of the following conditions: managed dyslipidemia, treated hypertension, diabetes mellitus under treatment, or active smoking. These participants had no clinical evidence of coronary artery disease, as confirmed by clinical evaluations, electrocardiograms, echocardiograms, and cardiac stress testing. Exclusion criteria for both study groups included any infection within one week prior to enrollment, immunosuppressed status, recent antibiotic use (within one month before participation), chronic viral infections, chronic inflammatory bowel disease, renal impairment (estimated glomerular filtration rate below 50 mL/min/1.73 m^2^), and pregnancy. Each participant provided written informed consent, permitting the use of their anonymized clinical information for research purposes. The study adhered to the principles outlined in the Declaration of Helsinki, with approval granted by the regional health authority (Agence Régionale de Santé-Midi-Pyrénées) and the local Ethics Committee.

### 2.2 Blood sampling and plasma levels of Rubicon determination

EDTA plasma samples were processed and frozen at −80 °C within 3 h of collection. Rubicon levels were determined using enzyme-linked immunosorbent assay (ELISA) kits (Human Rubicon ELISA Kit [HUFI06681], Assay Genie, Dublin, Ireland). The Rubicon assay follows a quantitative sandwich ELISA method, performed on EDTA plasma samples that were stored at −80 °C without being thawed (hemolyzed samples were excluded). After plating 100 μL of each sample, along with blanks and standards, a biotin-labeled primary antibody specific for Rubicon was added, followed by Avidin-conjugated Horseradish Peroxidase. The signal intensity, indicated by a yellow color, was detected at 450 nm using a microplate reader.

### 2.3 Statistical analysis

Quantitative data are expressed as means with standard deviations or medians with interquartile ranges (for Rubicon), while categorical data are presented as percentages. Comparisons of qualitative variables between the case and control groups were conducted using the Chi-squared (χ^2^) test, with Fisher's exact test applied when the assumptions of the χ^2^ test were not satisfied. Mean differences for quantitative variables were analyzed using the student's *t*-test. To assess normality and variance homogeneity, the Shapiro-Wilk test and Levene's test were employed, respectively. If the assumptions for the t-test were not met, data were either log-transformed or analyzed using the Wilcoxon-Mann-Whitney test (e.g., for age). Spearman's rank correlation, a non-parametric method, was used to examine the relationship between Rubicon levels and cardiovascular variables. Multivariate analysis was performed to evaluate autophagy-related factors, adjusting for potential confounders such as age, gender, HDL and LDL cholesterol, triglycerides, and glucose. Logistic regression models, including quadratic and cubic terms, were employed to explore potential non-linear relationships between continuous variables and case-control status. Due to the evident non-linearity with Rubicon, it was categorized based on its median for logistic regression. To assess Rubicon's discriminative ability between cases and controls, Receiver Operating Characteristic (ROC) curve analysis was performed, with the area under the curve (AUC) and its standard error calculated using Delong's method. All statistical tests were two-tailed with a significance threshold of 0.05. The analyses were carried out using SAS version 9.4 (SAS Institute, Cary, NC, USA) and STATA version 14.1 (StataCorp, College Station, TX, USA).

## 3 Results

### 3.1 Study population and measurement of plasma Rubicon concentrations

**Fig. 1:**
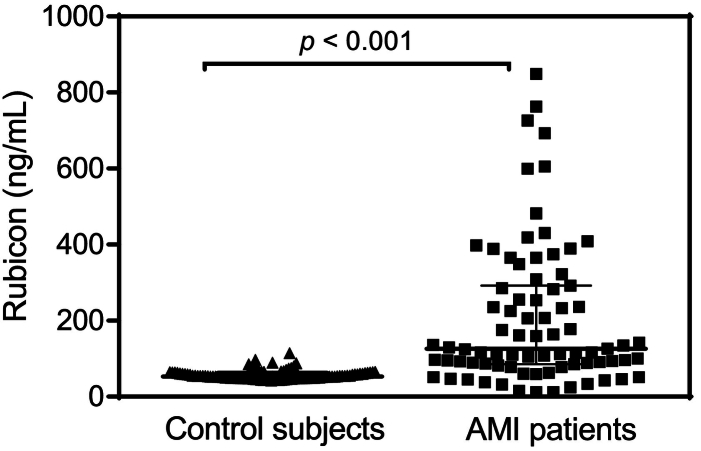
Plasma levels of Rubicon in the study population measured by enzyme-linked immunosorbent assay. The scatter dot plots represent the median values (pg/mL) with interquartile range. *p* value is considered statistically significant when <0.05.

The study included a total of 177 participants. Baseline characteristics of the myocardial infarction (MI) patients and control subjects are presented in Table 1. The control group, enrolled through the Cardiovascular Disease Prevention department, exhibited a high prevalence of hypertension, dyslipidemia, and obesity, as well as notable differences in medical treatments, reflecting their focus on preventive care. Plasma levels of Rubicon, a key regulator of autophagy, were quantified in the study population using an enzyme-linked immunosorbent assay (ELISA). The median [IQR] concentration of Rubicon was significantly higher in MI patients (126.5 pg/mL [85–291]) compared to control subjects (53 pg/mL [49–85]), with a *p*-value of <0.001 (Fig. 1).

**Table 1:** Baseline characteristics of the study population.

Variables		AMI patients (*n* = 79)	Control subjects (*n* = 98)	p-value
Sex (%)	Male	80	58	0.005
	Female	20	42	
Age (years)		64.9 (13.5)	60 (7.9)	0.016
Cardiovascular risk factors (%)				
Obesity		21.0	36.1	0.043
Dyslipidaemia		59.7	78.4	0.012
Diabetes		16.1	23.7	0.25
Hypertension		53.2	90.7	0.001
Current smoking		33.9	16.5	0.012
Heredity		37.5	37.1	0.77
Blood glucose (mg/dL)		130 (57)	110 (57)	0.039
Triglycerides (mg/dL)		120 (59)	135(71)	0.16
Total cholesterol (mg/dL)		189 (59)	199 (44)	0.21
LDL-cholesterol (mg/dL)		121 (54)	119 (40)	0.78
HDL-cholesterol (mg/dL)		47 (12)	53 (16)	0.016

Data are shown as % and mean with (standard deviation). LDL; Low-density lipoprotein, HDL; High-density lipoprotein.

### 3.2 Association between Rubicon levels and cardiovascular risk factors

**Fig. 2:**
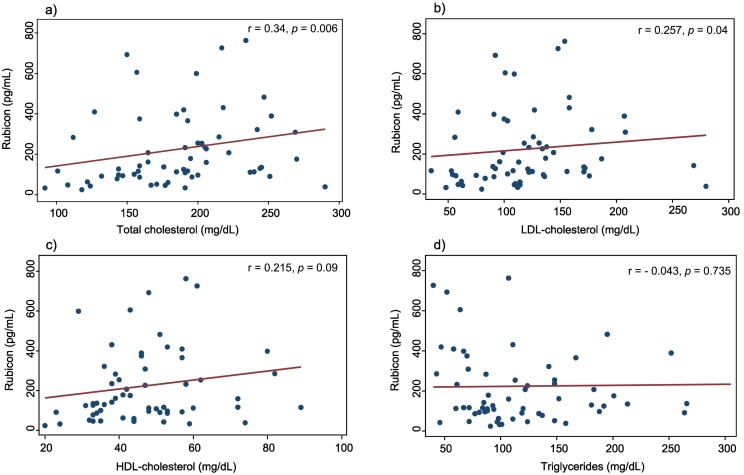
Scatter plots and Spearman's rank correlation coefficient between Rubicon and lipid parameters: total cholesterol (a), LDL-cholesterol (b), HDL-cholesterol (c) and triglycerides (d).

We performed correlation analyses to explore the relationship between Rubicon levels and cardiovascular risk factors. Spearman rank correlation coefficients indicated a significant positive association between Rubicon and lipid parameters in MI patients (Fig. 2). Specifically, Rubicon showed a notable correlation with total cholesterol (*r* = 0.257, *p* = 0.04) and LDL (low-density lipoprotein) cholesterol (*r* = 0.341, *p* = 0.006) but not with HDL (high-density lipoprotein) cholesterol (*r* = 0.215, *p* = 0.09) and triglycerides (*r* = − 0.043, *p* = 0.735).

### 3.3 Relationship between autophagy regulator Rubicon and myocardial infarction

The results presented in Table 2 show the multivariate analysis examining the relationship between the autophagy inhibitor Rubicon and the risk of MI. After adjusting for potential confounders including sex, age, LDL-cholesterol, HDL-cholesterol, glucose, and triglycerides, logistic regression revealed a significant link between elevated Rubicon levels and a higher risk of MI.

**Table 2:** Logistic regression analysis of rubicon as a risk factor for myocardial infarction.

	OR	95 % confidence limits		p value
Rubicon^a^	33.743	10.425	109.213	<0.0001

a Rubicon dichotomised (median), adjusted on sex, age, HDL-c, LDL-c, glucose and triglycerides.

### 3.4 ROC curve analysis and risk prediction for myocardial infarction using Rubicon

**Fig. 3:**
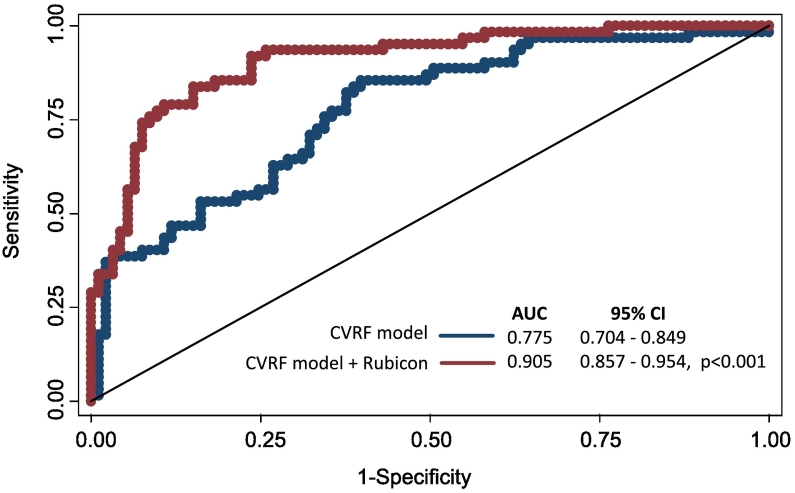
Receiver operating characteristic (ROC) curve analysis comparing the predictive value of the autophagy regulator Rubicon with a CVRF model (CVFR, cardiovascular risk factors) comprising sex, age, HDL-cholesterol, LDL-cholesterol, glucose, and triglycerides. AUC, area under the curve.

We assessed whether adding Rubicon to a cardiovascular risk factor (CVRF) model - consisting of age, sex, HDL-cholesterol, LDL-cholesterol, glucose, and triglycerides - would enhance its predictive accuracy. Receiver operating characteristic (ROC) curve analysis was conducted, and Fig. 3 displays the area under the curve (AUC) for both the clinical model alone and the model with Rubicon included. The integration of Rubicon significantly increased the model's predictive power, raising the AUC from 0.868 (95 % CI: 0.704–0.849) to 0.905 (95 % CI: 0.857–0.954), with a *p*-value of <0.001. These findings highlight the added predictive value of Rubicon as an autophagy-related biomarker when combined with conventional CVRF for forecasting MI.

## 4 Discussion

The identification of novel, reliable, and sensitive biomarkers for coronary artery disease remains a major clinical need. Our study investigated the circulating levels of Rubicon, a key autophagy regulator, in relation to MI and explored its potential as a novel biomarker for predicting MI risk. The findings highlight significant differences in plasma Rubicon concentrations between MI patients and control subjects, as well as its associations with lipid cardiovascular risk factors. Additionally, we demonstrated that incorporating Rubicon into traditional cardiovascular risk models improves their predictive accuracy for MI. Elevated plasma Rubicon concentrations observed in MI patients compared to control subjects (126.5 pg/mL vs. 53 pg/mL, *p* < 0.001) suggest that Rubicon may play a significant role in the pathophysiology of MI. However, our previous findings [22] showed that Rubicon was inversely associated with acute coronary syndrome risk, which contrasts with the current results showing elevated Rubicon levels in MI patients. Several potential explanations could reconcile these contrasting observations. Elevated Rubicon levels in MI patients might reflect a maladaptive response to ischemic injury, where excessive inhibition of autophagy leads to impaired cellular repair and heightened vulnerability to myocardial damage. This supports the idea that dysregulated autophagy, either too much or too little, can contribute to MI risk. Additionally, when comparing the mean age of the MI patients (64.9 years), they are older than the patients (57.46 years) in our previous study [22]. This observation aligns with previous studies showing that, as organisms age, Rubicon levels naturally increase, contributing to the decline in autophagic activity observed in older individuals. As demonstrated, the knockdown of Rubicon extends the lifespan of worms and flies and ameliorates several age-associated phenotypes [26]. The correlation between Rubicon and lipid parameters further supports its potential link to cardiovascular risk. Our analyses revealed that Rubicon levels positively correlated with total cholesterol and LDL cholesterol in MI patients, suggesting a possible interaction between autophagy dysregulation and lipid metabolism in the progression of atherosclerosis. Our findings are consistent with studies demonstrating downregulation of autophagy in the heart tissues of hypercholesterolemic rats [27]. Elevated LDL cholesterol is a well-known risk factor for atherosclerotic plaque formation, and the observed association with Rubicon may indicate that dysregulated autophagy could exacerbate plaque vulnerability, contributing to plaque rupture and the subsequent onset of MI. Interestingly, Rubicon did not correlate significantly with HDL cholesterol or triglycerides, indicating that Rubicon levels may be associated with specific lipid components, potentially tied to more atherogenic lipid types like LDL. This suggests that Rubicon levels may be associated with specific lipid components. The lack of association with triglycerides and HDL cholesterol might suggest that Rubicon's involvement in lipid metabolism could be specific to certain pathways, perhaps those linked to cholesterol transport or storage that are regulated by autophagy rather than reverse cholesterol transport and triglyceride metabolism. Multivariate analysis confirmed that elevated Rubicon levels were independently associated with an increased risk of MI, even after adjusting for traditional cardiovascular risk factors, including age, sex, LDL-cholesterol, HDL-cholesterol, glucose, and triglycerides. This suggests that Rubicon may serve as a valuable biomarker for MI risk, beyond the established clinical markers. The most compelling finding of this study is the improvement in predictive performance when Rubicon is incorporated into conventional risk models for MI. The ROC curve analysis demonstrated a significant increase in the AUC from 0.868 to 0.905 (*p* < 0.001) when Rubicon was added to a model that included sex, age, LDL-cholesterol, HDL-cholesterol, glucose, and triglycerides. Our result underscores the incremental value of Rubicon as a biomarker, suggesting that it provides unique information about MI risk that is not captured by traditional cardiovascular risk factors alone. The improved predictive accuracy highlights Rubicon's potential utility in clinical practice, where it could enhance risk stratification and guide more personalized therapeutic decision-making.

However, several limitations must be acknowledged. This is a case-control study, and longitudinal data are needed to establish the temporal relationship between Rubicon levels and MI onset. Additionally, while the association between Rubicon and lipid parameters is intriguing, further research is required to understand the underlying mechanisms driving these correlations and their clinical significance. We focused our study exclusively on Type MI patients, which precludes the assessment of Rubicon's potential correlation with cardiac troponin levels across different types of MI, such as Type 2 MI. Measuring Rubicon levels in future studies that include both Type 1 and Type 2 MI patients could provide valuable insights into its potential as a biomarker to distinguish between these two types of MI. Including measurements of Rubicon levels in healthy subjects would be also interesting, as it could establish a baseline reference, enabling a clearer understanding of how Rubicon levels are altered in cardiovascular risk and MI, and potentially uncovering its role in early disease processes.

Further studies are needed to clarify whether the differential expression of circulating Rubicon is due to altered intracellular production, distinct release mechanisms, or a combination of both. Notably, a recent study demonstrated that stressed cardiomyocytes can release large extracellular vesicles derived from autophagic processes, termed ‘exopheres,’ which contain mitochondria and sarcomere proteins [28]. This raises the intriguing possibility that autophagy-mediated exocytosis could contribute to the release of proteins like Rubicon into the circulation.

## 5 Conclusions and future directions

The identification of Rubicon as a circulating biomarker for MI opens new avenues for both diagnostic and therapeutic interventions. First, measuring plasma Rubicon levels could aid in the early detection of individuals at high risk for MI, particularly when used alongside traditional risk factors. Second, the role of Rubicon in autophagy regulation suggests that modulating its activity could be a potential therapeutic target for reducing ischemic injury in MI patients. Pharmacological strategies aimed at restoring autophagic balance, either by inhibiting Rubicon or enhancing autophagy flux, may provide novel treatment options for mitigating myocardial damage and improving outcomes in MI patients.

## CRediT authorship contribution statement

**Marie-Hélène Grazide:** Investigation, Data curation. **Jean-Bernard Ruidavets:** Writing – review & editing, Methodology, Formal analysis. **Wim Martinet:** Writing – review & editing, Funding acquisition. **Meyer Elbaz:** Validation, Supervision, Project administration, Methodology, Funding acquisition. **Cécile Vindis:** Writing – review & editing, Writing – original draft, Validation, Project administration, Methodology, Funding acquisition, Formal analysis, Data curation, Conceptualization.

## Informed consent statement

All patients and participants provided written informed consent prior to their participation in the study.

## Institutional review board statement

The study (DC-2008-4623, NCT02405468) was conducted in accordance with the Declaration of Helsinki, reviewed and approved by the Agence Régionale de Santé Occitanie-Midi-Pyrénées (Toulouse, France) and the Institutional Ethics Committee for Human Research, Comité de Protection des Personnes du Sud-Ouest (Toulouse, France).

## Funding

This research was funded by an INSERM International Research Project (SUBSIDI) awarded to Dr. C. Vindis and Pr. W Martinet, as well as by “La Fédération Française de la Cardiologie,” which supported Dr. C. Vindis and Pr. M. Elbaz.

## Declaration of competing interest

The authors declare that they have no known competing financial interests or personal relationships that could have appeared to influence the work reported in this paper.
